# TRAIL DR5-CTSB crosstalk participates in breast cancer autophagy initiated by SAHA

**DOI:** 10.1038/cddiscovery.2017.52

**Published:** 2017-08-21

**Authors:** Han Han, Hui Zhou, Jing Li, Xiuyan Feng, Dan Zou, Weiqiang Zhou

**Affiliations:** 1 Key Laboratory of Environmental Pollution and Microecology of Liaoning Province, Shenyang Medical College, No.146 North Huanghe St, Huanggu Dis, Shenyang City, Liaoning Pro 110034, China; 2 Department of Biochemistry and Molecular Biology, Shenyang Medical College, No.146 North Huanghe St, Huanggu Dis, Shenyang City, Liaoning Pro 110034, China

## Abstract

To investigate the ability of SAHA-induced TRAIL DR5-CTSB crosstalk to initiate the breast cancer autophagy, RTCA assay was performed to assess the effect of SAHA on breast cancer cells, and western blot and ELISA were used to verify the inductive effects on expression of CTSB. Breast cancer cells were transfected with TRAIL DR5 siRNA to block the function of TRAIL DR5. Cell viability and apoptosis of breast cancer cells were analyzed using a muse cell analyzer. The distribution of LC3-II in TRAIL DR5-silenced breast cancer cells treated with SAHA was observed by immunofluorescence microscopy, the mRNA levels of autophagy-related genes were detected by RNA microarray, and the activity of autophagy-related signaling pathways was screened by MAPK antibody array. Results indicated that SAHA did indeed repress the growth of breast cancer cell lines with inducing CTSB expression. Western blot and ELISA results indicated that TRAIL DR5 was involved in the expression of CTSB in SAHA-induced breast cancer cells. Cell viability and apoptosis assays showed that the inactivation of TRAIL DR5 can significantly inhibit the effects of SAHA. An immunofluorescence assay indicated that, with SAHA treatment, MDA-MB-231 and MCF-7 cells underwent apparent morphological changes. While SAHA was added in the TRAIL-DR5 blocked cells, the distribution of LC3-II signal was dispersed, the intensity of fluorescence signal was weaker than that of SAHA alone. RNA array indicated that SAHA significantly increased mRNA expression of autophagy marker LC3A/B whereas the change was significantly reversed in TRAIL DR5-silenced cells. The results of MAPK antibody array showed that SAHA and TRAIL DR5 could affect the activity of AKT1, AKT2, and TOR protein in breast cancer cells. These results provide more evidence that SAHA may stimulate TRAIL DR5-CTSB crosstalk, influence the activity of downstream TOR signalling pathway mainly through the AKTs pathway, and initiate the autophagy of breast cancer cells.

## Introduction

Breast cancer has a serious impact on women’s health and it can be life-threatening. Recent data show that the United States is projected to see 1.69 million new cancer patients and nearly 600 000 deaths in 2017, of which 253 500 new cases will be breast cancer in women. The incidence of breast cancer has become the highest of any type of cancer, and its mortality rate is about to reach second in women.^1^

Despite the lack of clear understanding of its pathogenesis, breast cancer is known to be a hormone-dependent carcinoma in which carcinogenesis is closely associated with the abnormality of related oncogenes and anti-oncogenes.^2^ In recent years, the well-researched development of epigenetics has shown that suberoylanilide hydroxamic acid (SAHA, vorinostat), a histone deacetylase inhibitor (HDACi), has strong anti-tumor activity. It can bind to the specific lysine residues in core histone N-terminal and remove the hydrophobic acetyl groups, thereby inhibiting the transcription of some of the genes responsible for cell proliferation, differentiation, and apoptosis.^3,4^ Because of its good effects in the pre-clinical observations, SAHA has broad prospects for application.

Tumor-necrosis-factor-related apoptosis-inducing ligand death receptor 5 (TRAIL DR5) is a transmembrane receptor of the tumor necrosis factor (TNF) superfamily. It can activate TRAIL-induced apoptosis in a variety of cancer cells.^5–8^ Studies have also shown that TRAIL DR5 can trigger autophagy-related pathways and cause cell death.^9–12^

The process of autophagy was first observed by Ashford and Porter in 1962, when they discovered the phenomenon of autolytic cell destruction.^13,14^ For cancer cells, autophagy is a double-edged sword. The lower intensity of autophagy response to cancer cells is beneficial to cell survival and proliferation. However, if the cell autophagy is intense or long-lasting, it can induce the type II programmed cell death (PCD) to the cancer cells.^15,16^

The occurrence of autophagy is closely related to lysosomes. Lysosomal cathepsins, which are enclosed in the lysosomes, play important roles in cell death.^17,18^ Cathepsin B (CTSB) is the first cysteine protease found to be associated with breast cancer. The mature CTSB, with a heavy chain of 25 kDa and a light chain of 5 kDa, has peptide hydrolase and endonuclease activities.^19,20^

CTSB plays a dual role in breast carcinogenesis. First of all, CTSB is involved in the initiation, regulation, and termination of a variety of biological molecules. These molecules interact closely with DNA replication, cell cycle progression, and differentiation. However, when lysosomal membrane integrity is damaged by the drugs or other factors, a large volume of CTSB, beyond the normal metabolic requirements for the cell, is extravasated from lysosomes. CTSB can have harmful effects including cell autophagy.^21–24^

Although SAHA has good clinical prospects, a large number of laboratory studies and clinical applications have also exposed some shortcomings, such as its excessive toxicity at high doses, tendency to metabolize, short *in vivo* half-life, and susceptibility to resistance in response to long-term use. For this reason, it is highly necessary to screen new targets of SAHA for better efficacy. In this study, breast cancer ER-positive cell MCF-7 and ER-negative cell MDA-MB-231 are selected to investigate the effects of SAHA on TRAIL DR5-CTSB crosstalk to initiate the breast cancer autophagy.

## Results

### SAHA inhibits the growth of breast cancer cells

**Figure 1:**
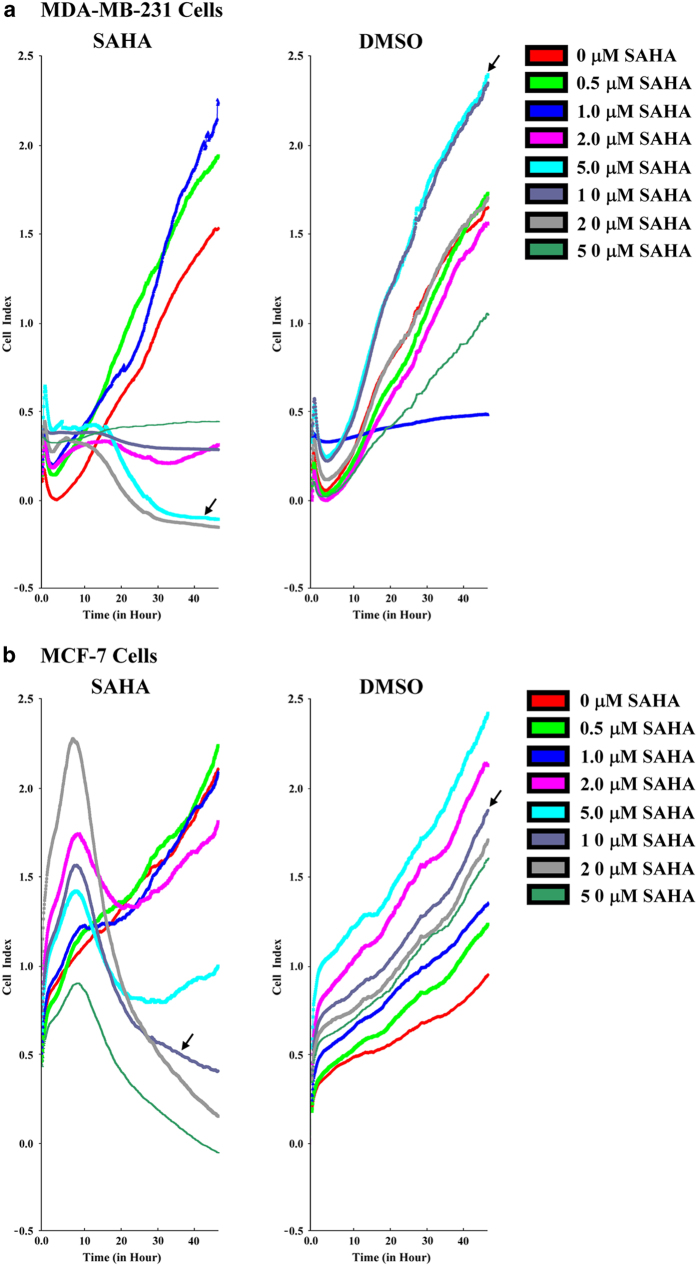
The effects of SAHA on breast cancer cells. MDA-MB-231 (**a**) or MCF-7 (**b**) cells were incubated with various concentrations of SAHA (0, 0.5, 1.0, 2.0, 5.0, 10, 20, and 50 *μ*M) for 24 h. RTCA assay was used to monitor the effect of SAHA on breast cancer cells. DMSO was treated as control.

To investigate the effect of SAHA on breast cancer cells, we measured the cell prolifertion in breast cancer MDA-MB-231 and MCF-7 cells with different concentrations of SAHA (0–50 *μ*M) using RTCA assay. The results showed that, although it did not show a dose-dependent response, 5 *μ*M SAHA got the optimal inhibitory effect with minimal toxicity in MDA-MB-231 cells, and 10 *μ*M SAHA in MCF-7 cells (Figures 1a and b).

We also compared the effects of DMSO on MDA-MB-231 and MCF-7 cells. As shown in Figure 1, the cell index curves did not change significantly with DMSO treatment for the same concentrations of SAHA, which indicated that DMSO had no pronounced effects on the breast cancer cells.

### The activity of TRAIL DR5 is involved in SAHA-induced expression of CTSB in breast cancer cells

**Figure 2:**
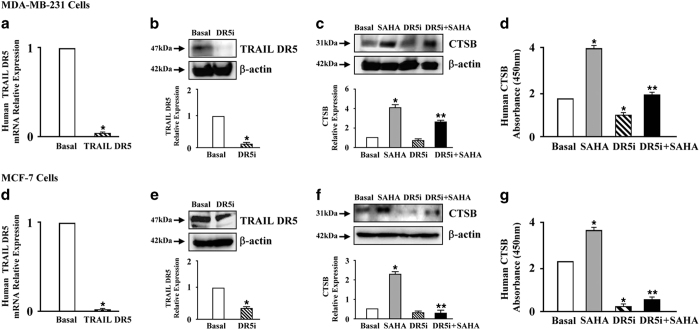
The effects of SAHA and TRAIL DR5 on CTSB expression. After starvation, 50% confluent MDA-MB-231 or MCF-7 cells plated in 6-well plate were transfected with 10 *μ*M TRAIL DR5 siRNA targeting human TRAIL DR5 using lipofectamine 3000 reagent according to the manufacturer’s protocol. Then the cells were incubated with 5 *μ*M SAHA for 24 h. Real-time PCR (**a** and **d**), Western blot (**b**, **c**, **e** and **f**) and ELISA (**d** and **g**) were used to access the expression of TRAIL-DR5 or CTSB. Relative quantitative values represent mean±S.E.M.; * represents statistical significance of *P*<0.05 comparing with Basal, ** represents statistical significance of *P*<0.05 comparing with SAHA.

Next, we determined whether TRAIL DR5 regulates SAHA-induced CTSB expression in breast cancer cells. TRAIL DR5 siRNAs were transfected into MDA-MB-231 and MCF-7 cells to block TRAIL DR5 function. Real-time PCR and western blot results were confirmed that the function of TRAIL DR5 was mostly blocked by the addition of specific siRNA in both breast cancer cell lines (Figures 2a, b, d and e).

Next, the changes in the levels of CTSB protein in breast cancer cells were assessed. The results of western blot analysis showed that SAHA alone significantly increased the expression of CTSB in MDA-MB-231 and MCF-7 cells, while blocking the function of TRAIL DR5 alone did not significantly inhibit the expression of CTSB. However, when TRAIL DR5-silenced cells were treated with SAHA, the expression of CTSB was significantly inhibited (Figures 2c and d). The results of ELISA confirmed the results of western blot analysis (Figures 2f and g). These results indicate that SAHA-induced CTSB expression requires participation of TRAIL DR5.

### The effect of SAHA and TRAIL DR5 on the cell viability and apoptosis of breast cancer cells

**Figure 3:**
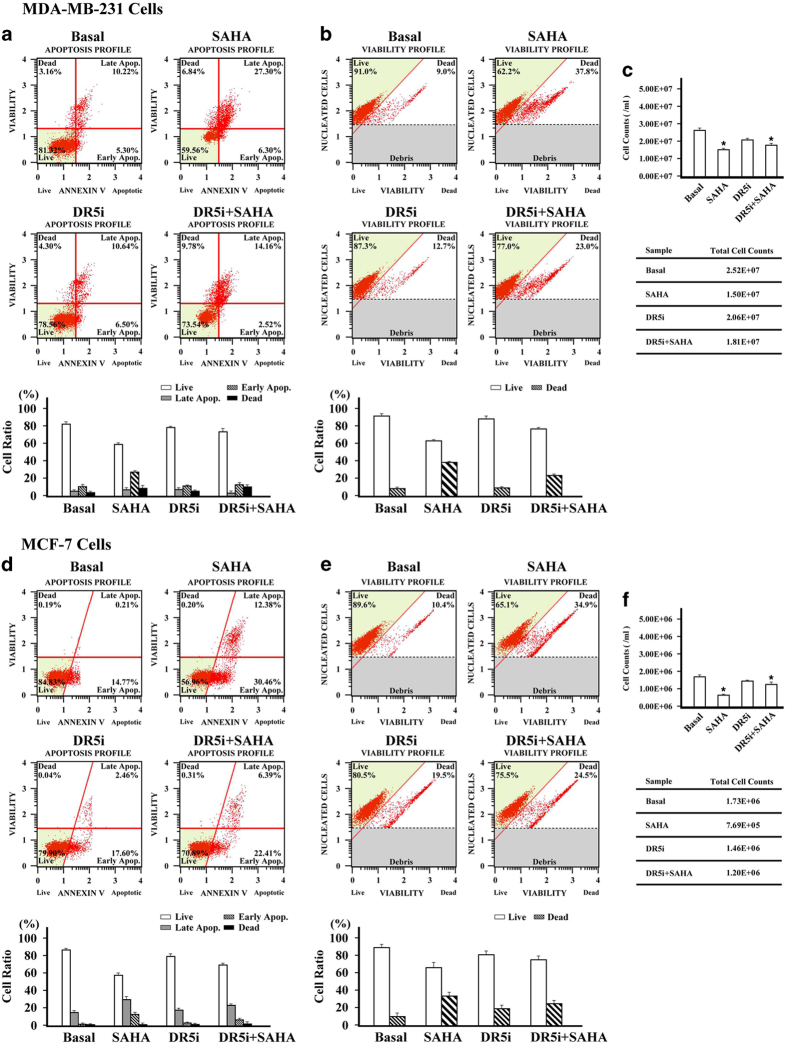
The effects of SAHA and TRAIL DR5 on cell viability and apoptosis of cancer cells. After transfected by TRAIL DR5 siRNA, MDA-MB-231 or MCF-7 cells were incubated with either 5 *μ*M or 10 *μ*M SAHA for 24 h, respectively. Fifty milliliters cell suspension from 2×10^5^ of collected cells was added with 450 *μ*l count and viability reagent. Muse count and viability software module was used to analyze the results. For the apoptotic assay, 100 *μ*l of Muse annexin V and dead cell reagent was added with 1×10^6^ cells for 20 min at room temperature. Muse cell analyzer was used to determine the percentages of alive, apoptosis, and dead cells. (**a**) The apoptosis profile in MDA-MB-231. (**b**) The cell viability profile in MDA-MB-231. (**c**) The cell count in MDA-MB-231. (**d**) The apoptosis profile in MCF-7. (**e**) The cell viability profile in MCF-7. (**f**) The cell count in MCF-7. Relative quantitative values represent mean±S.E.M.; * represents statistical significance of *P*<0.05 comparing with Basal.

In order to further clarify the role of SAHA and TRAIL DR5 in the inhibition of breast cancer growth, we analyzed viability and apoptosis in breast cancer cells. Results showed the growth of breast cancer cells to be significantly inhibited by SAHA alone: the number of cells decreased, and cell viability decreased significantly. Silencing of TRAIL DR5 function had little effect on breast cancer cells: neither the total number of cells nor cell viability changed significantly. When SAHA was treated with TRAIL-DR5-silenced cells, cell viability was recovered to a visibly greater extent than with SAHA alone. The live cell ratio in MDA-MB-231 and MCF-7 cells increased from 62.2 to 77.0% and from 65.1 to 75.5%, respectively (Figures 3b, c, e and f). Similar results were observed in an apoptosis assay. The ratio of live cells in TRAIL-DR5-silenced MDA-MB-231 cells treated with SAHA increased from 59.56% in the control group to 73.54%. The percentage of apoptotic cells (including early and late phase apoptosis) and dead cells decreased from 40.44% with SAHA alone to 26.46% (Figures 3b and e). In MCF-7 cells, the ratios of live cells went from 56.96 to 70.89% (Figures 3a and d). These results indicate that the inactivation of TRAIL DR5 can significantly inhibit the effect of SAHA on breast cancer cells.

### SAHA and TRAIL DR5 activates the expression of autophagy marker LC3-II in breast cancer cells

**Figure 4:**
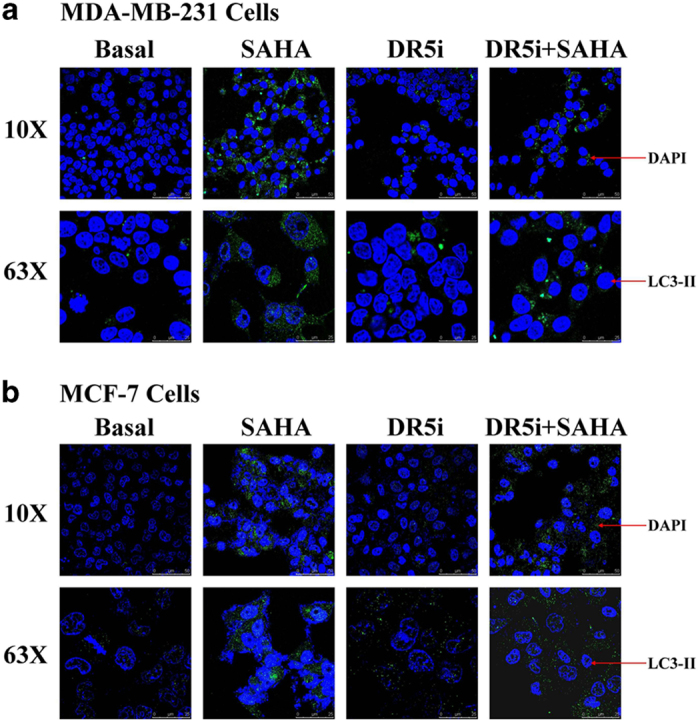
LC3-II immuno-fluorescence assay induced by SAHA and TRAIL DR5. TRAIL DR5 transfected cells of MDA-MB-231 (**a**) or MCF-7 (**b**) were incubated with SAHA in 6-well plate. The cells were fixed with 4% formaldehyde at room temperature and blocked with buffer solution for 2 h at room temperature. LC3-II antibody solution was added to incubate with cells overnight at 4 °C. A DyLight 488 fluoresence antibody (1 : 200 dilution) was used for 1 h incubation and nuclei were counterstained with DAPI dye for another 10 min. The cells were imaged and autophagy signals were visualized by Leica DMI6000 B microscope with ×10 (scale bars indicate 50 *μ*m) and ×63 (scale bars indicate 25 *μ*m) magnification.

To determine whether autophagy was involved in SAHA and TRAIL DR5 treatment in breast cancer cells, we assessed the expression of autophagy marker LC3-II by immunofluorescence assay. From the results shown in Figure 4, the cell morphology in untreated controls was normal, and cell growth was good. After 24 h incubation, the cell density reached 90% confluence. LC3-II fluorescence distribution was diffused and weak and did not express any aggregation. After SAHA treatment, MDA-MB-231 and MCF-7 cells showed apparent morphological changes. The cells were rounded, shrunken, broken, or acircular, and the granules in the cells were increased. LC3-II fluorescence showed a spot-like distribution, and also there were a number of lamellar bright green fluorescence aggregates within the cytoplasm. Blocking the activity of TRAIL DR5 did not render the cell morphology or LC3-II fluorescence expression of breast cancer cells visibly different from those of control cells. However, when SAHA was added to the TRAIL-DR5-blocked cells, only a small amount of green fluorescence was observed. The distribution of LC3-II signal was dispersed, the intensity of fluorescence signal was weaker than that of SAHA alone.

### The effect of SAHA and TRAIL DR5 on the expression of autophagy-related molecules in breast cancer cells

**Figure 5:**
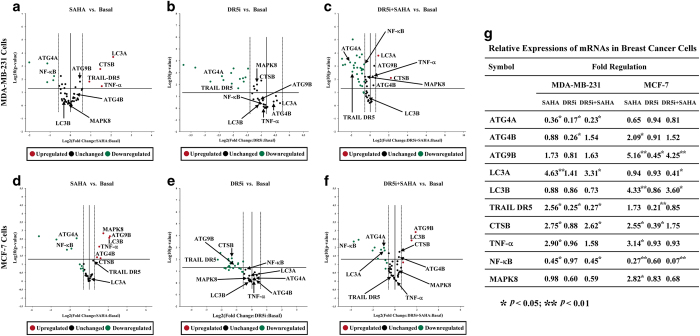
The effects of SAHA and TRAIL DR5 on the expressions of autophagy-related genes in cancer cells. RNA was isolated from MDA-MB-231 or MCF-7 cells using high pure RNA isolation kit following manufacturer’s instructions. To obtain the first-strand cDNA, transcriptor first strand cDNA synthesis kit was used and cDNA was as a template in real-time PCR reactions with power SYBR green PCR master mix. Exprofile human autophagy Gene qPCR array kit was employed to describe related mRNA expression. Relative changes of gene expression in the array were calculated using the 2^−ΔΔCt^ (threshold cycle) method. (**a**–**c**). MDA-MB-231 cells, (**d**–**f**). MCF-7 cells, (**g**). Related data analysis. Relative quantitative values represent mean±S.E.M.; * represents statistical significance of *P*<0.05 comparing with Basal, ** represents statistical significance of *P*<0.01 comparing with Basal.

To further elucidate the specific pathways of autophagy induced by SAHA and TRAIL DR5, the mRNA expressions of autophagy-related genes in TRAIL-DR5-silenced breast cancer cells treated with SAHA were detected by RNA microarray. As shown in Figure 5, SAHA significantly increased mRNA expression of autophagy marker LC3A in MDA-MB-231 and LC3B in MCF-7 cells, whereas the expression was reversed in TRAIL-DR5-silenced cells. In addition, the results showed that SAHA stimulated the expression of ATG9B in both groups of cells, despite slightly different effects in the cell lines. In addition, levels of ATG4B were not changed significantly in MDA-MB-231, but there had a raising effect of ATG4B with SAHA induction in MCF-7 cells.

**Figure 6:**
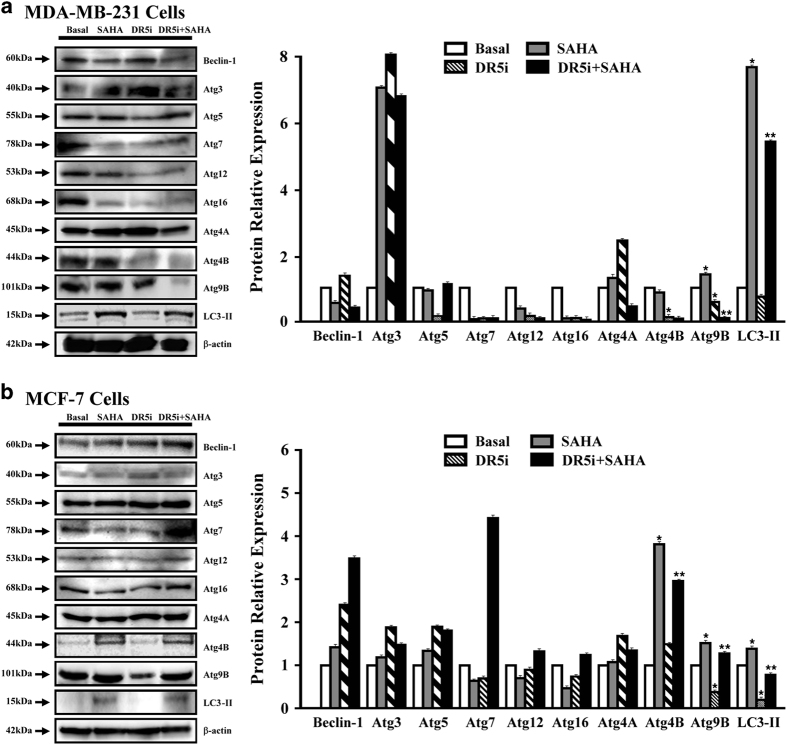
The effects of SAHA and TRAIL DR5 on the expressions of autophagy-related proteins. The proteins were extracted from TRAIL DR5 siRNA transfected MDA-MB-231 (**a**) or MCF-7 (**b**) cells treated with SAHA. Western blot analysis was used to access the levels for autophagy-related factors. β-actin was used as loading controls. Protein bands were detected using Supersignal west pico plus chemiluminescent substrate and exposed on DNR MF-Chemi Bio-imaging systems. Relative quantitative values represent mean±S.E.M.; * represents statistical significance of *P*<0.05 comparing with Basal, ** represents statistical significance of *P*<0.05 comparing with SAHA.

Then we used western blot analysis to detect the protein expressions of autophagy-related factors. From the results shown in Figure 6, in MDA-MB-231 cells, the levels of Beclin-1, ATG5, ATG7, ATG12, ATG16, ATG4A, and ATG4B had not been activated effectively with SAHA treatment. Although the production of ATG3 was enhanced obviously with SAHA or TRAIL DR5 siRNA transfection, the combination treatment did not repress the expression of ATG3. TRAIL DR5 siRNA transfection can decrease the expression of ATG4B, but SAHA had no activated effect on the ATG4A expression clearly. SAHA can increase the expression of ATG9B and LC3-II in MDA-MB-231 cells, and this activating effect was depressed after TRAIL DR5 siRNA transfection. In MCF-7 cells, similarly, the expressions of Beclin-1, ATG3, ATG5, ATG7, ATG12, ATG16, and ATG4A can not be activated effectively with SAHA treatment. However, SAHA activated ATG4B, ATG9B and LC3-II expressions and the increases were repressed after blockage of TRAIL DR5 activity.

### TOR signaling pathways regulate autophagy activated by SAHA and TRAIL DR5 in breast cancer cells

**Figure 7:**
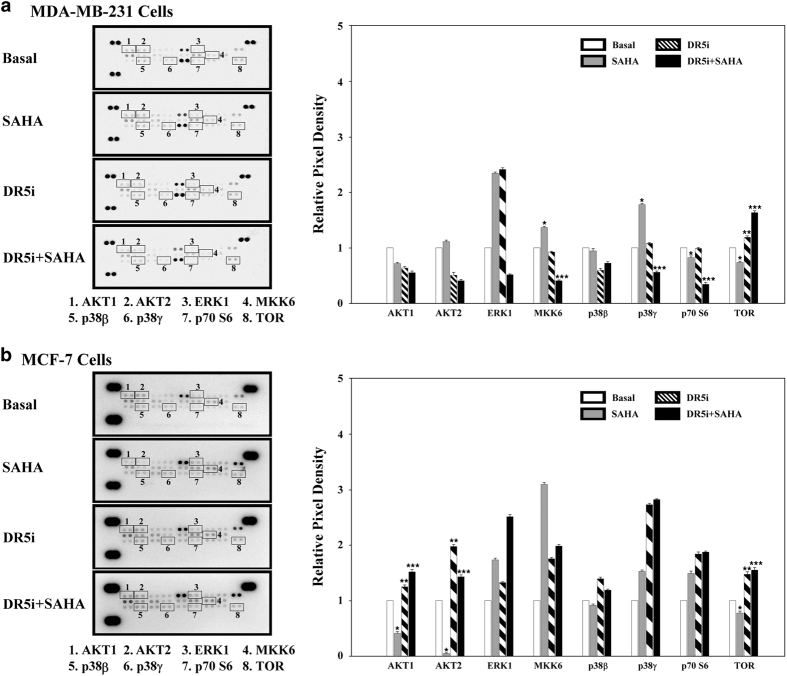
The screening of autophagy-related signaling pathways involved in the treatment with SAHA and TRAIL DR5. First, ~2×10^7^ cells with SAHA and TRAIL DR5 siRNA treatment were solubilized in lysis buffer. The lysates were resuspended and protein concentrations of the resulting lysates were measured using a BCA protein assay kit. MAPK antibody array was used to screen the activity changes involved in autophagy-related signaling pathways. The membrane intensity was acquired using chemiluminescence and pixel densities can be analyzed using Gelpro analyzer software. Densities were measured as a percentage of the positive controls included on each membrane. (**a**) MDA-MB-231 cells. (**b**) MCF-7 cells. Relative quantitative values represent mean±S.E.M., * represents statistical significance of *P*<0.05 comparing with Basal, ** represents statistical significance of *P*<0.05 comparing with Basal, *** represents statistical significance of *P*<0.05 comparing with TRAIL DR5.

**Figure 8:**
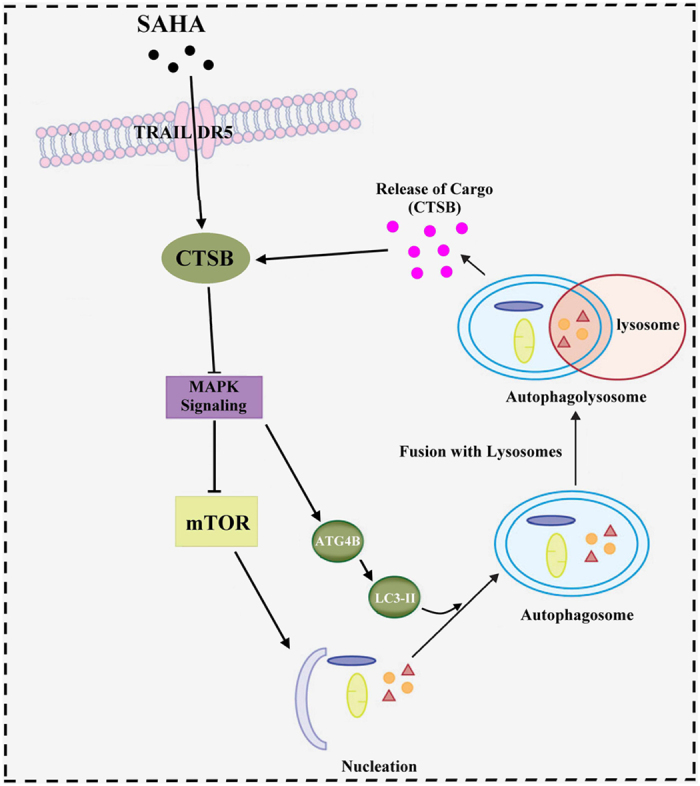
The proposed model of the cell autophagy induced by SAHA and TRAIL DR5-CTSB in breast cancer cell lines. TRAIL DR5 was involved in SAHA-induced autophagy with up-regulation of CTSB. Activity changes of MAPK and TOR signaling pathway were involved in the response to SAHA and TRAIL DR5. As important regulators, ATG4B and LC3-II functioned as targets and facilitated the release of engulfed cargo in autophagolysosome.

In order to further understand the mechanisms underlying SAHA- and TRAIL-DR5-induced autophagy in breast cancer cells, we used MAPK antibody array to screen the activity changes involved in autophagy-related signaling pathways. Results, such as those shown in Figure 7 and 8, SAHA alone significantly inhibited the expression levels of TOR proteins in MDA-MB-231 and MCF-7 cells but TRAIL DR5 increased its expression in both cell lines. Also, while SAHA treatment in TRAIL DR5-silenced cells, the production of TOR was obviously gone up again. Interestingly, SAHA and TRAIL DR5 could affect the expressions of AKT1 and AKT2 in MCF-7 cells, and had no clear effects in MDA-MB-231 cells. Instead, p70S6 expression was influenced mainly in MDA-MB-231 cells. These results suggest that SAHA and TRAIL DR5 may induce autophagy through TOR signal transduction pathways, and AKTs might play some regulatory role in the process, especially in ER-positive breast cancer cell line.

## Discussion

Cell autophagy essentially consists of the following stages. First, a bilayer membrane structure from impending autophagic cells is shredded to form pre-autophagic vacuoles. Then the vacuoles take in degraded organelles and other components to form autophagosomes. Finally, the outer membrane of each autophagosome fuses with the lysosomal membrane to form autophagolysosomes. Autophagolysosome utilizes various proteases involved in the vacuole to degrade contained substances for the metabolic needs.^25–28^

Cell autophagy-induced cell death is highly different from cell apoptosis and necrosis. It is characterized by the early organelle degradation, no caspase activation, and the continued integrity of DNA and the cytoskeleton. In addition, the lack of response to the infectious inflammation also distinguishes autophagy-induced cell death from cell necrosis.^29,30^ Exploring the mechanisms by which autophagy has important significance for breast cancer treatment and prevention.

In this study, results showed that SAHA could significantly inhibit the growth of breast cancer cells, resulting in a decrease in total cell counts and cell viability. Western blot analysis showed that the protein expression of CTSB was increased in SAHA-treated breast cancer cells. Our previous studies have demonstrated that CTSB plays an important role in SAHA-induced autophagy of breast cancer cells. Abnormal elevation of CTSB level can be used as a biomarker to anticipate the autophagy in breast cancer cells.^31^ In addition, SAHA can initiate the apoptosis of breast cancer cells through TRAIL DR5.^32^ Because apoptosis and autophagy are two closely related biological processes within cells and because there is not much evidence to indicate whether SAHA induces autophagy through TRAIL DR5-CTSB, we used specific siRNA to block the function of TRAIL DR5 and explored the roles of TRAIL DR5 in SAHA-induced autophagy.

The expression of CTSB was not activated by SAHA after TRAIL DR5 blockage, and the relative expression of CTSB in mRNA and protein levels was significantly decreased, indicating that TRAIL DR5 is required to activate SAHA-induced CTSB expression.

As a specific marker of autophagy, the newly synthesized LC3 precursor protein is cut by autophagy-related protein 4 (ATG4) to expose its glycine residue in the C terminus. LC3 is conjugated to phosphatidylethanolamine (PE) to form autophagosomes.^33–36^ In the study, we used immunofluorescence microscopy to observe the distribution of LC3-II in breast cancer cells. The morphological changes underlying autophagy are shown in MDA-MB-231 and MCF-7 cells treated with SAHA. The fluorescence spots of LC3-II showed a patchy distribution, the fluorescence signal was obvious and the cytoplasm had a number of flaky bright fluorescence aggregates. However, only a small number of MDA-MB-231 and MCF-7 cells showed accumulation of green fluorescence with SAHA treatment in TRAIL-DR5-silenced cells, and the distribution of LC3-II signal was decreased and the intensity of fluorescence signal was weaker than that of SAHA alone. These results further demonstrate that the autophagy by SAHA induction requires TRAIL DR5 activation in breast cancer cells.

The role of ATG4B at the late stage of autophagolysosome formation is essential. ATG4B clears ATG8 from the autophagosome membrane by delipidation, whereas this process is necessary for the formation of autophagolysosome.^37–40^ We found that SAHA significantly increased the expression levels of LC3A/B mRNA and protein in MDA-MB-231 and MCF-7 cells, but the induction effects on ATG4B were different. In MDA-MB-231 cells, SAHA effected not significant stimulation on ATG4B expression, but it vigorously enhanced ATG4B expression in MCF-7 cells.

Interestingly, despite the lack of clear induction on ATG4B activity, SAHA was found to activate ATG9B overexpression in MDA-MB-231 cells. Because ATG9B has a similar spatial conformation with ATG4B, it is here hypothesized that ATG9B may process LC3 in the absence of ATG4B under some certain conditions. It is noteworthy that TRAIL DR5 siRNA transfection also inhibits the induction effects of SAHA on the proteins. We here concluded that autophagy induced by SAHA in breast cancer cells is involved in TRAIL DR5, and this induction may be related to the increased catalytic activity of ATG4B (or other ATG molecules with similar functions, such as ATG9B).

MAPKs (Mitogen-activated protein kinases), which are activated by many different signals, direct the execution of appropriate genetic programs, including activation of gene transcription, protein synthesis, cell cycle machinery, cell death, and differentiation. We used solid-phase MAPK antibody array in the study in order to elucidate the related signaling pathways (PI3K/ATK, ERK, AMPK, and p53) involved in the process of autophagy induced by SAHA and TRAIL DR5. The results showed that SAHA and TRAIL DR5 could regulate autophagy by influencing the activities of TOR protein in breast cancer cells.

We found that SAHA has different effects on ER-positive and negative breast cancer cells. This point focuses on the induction effects of SAHA on AKTs expressions. The results of SAHA treatment in TRAIL DR5-silenced MCF-7 cells, a ER-positive breast cancer cell line, displayed that AKT1 and ATK2 play important regulatory roles in SAHA-TRAIL DR5 induced autophagy, but AKTs had slight effects on monitoring autophagy in ER-negative cells such as MDA-MB-231.

TOR is an endogenous inhibitory center of autophagy, and p70S6 is an important substrate kinase for TOR. If the activity of p70S6 protein kinase is reduced, autophagy can be initiated directly.^41,42^ In this way, although the effects of SAHA and TRAIL DR5 on two cell lines were slightly different, the present study showed that SAHA and TRAIL DR5 may influence the activity of downstream TOR signaling pathway mainly through MAPK pathway, especially AKTs, and so induce autophagy in breast cancer cells.

## Materials and Methods

### Cell lines and reagents

SAHA was from Sigma-Aldrich (St Louis, MO, USA). Lipofectamine 3000 reagent was from Thermo Fisher Scientific (Waltham, MA, USA). Muse cell cycle kit, Muse annexin and dead cell kit, and Muse count and viability kit were from Millipore (Darmstadt, Germany). Human MAPK antibody array kit was purchased from R&D Systems (Minneapolis, MN, USA). High pure RNA isolation kit and transcriptor first strand cDNA synthesis kit were obtained from Roche Diagnostics GmbH (Mannheim, Germany). Exprofile human autophagy gene qPCR array kit was obtained from Genecopoeia (Rockville, MD. USA). Power SYBR green PCR master mix, RIPA cell lysis buffer and BCA protein assay kit were from Life Technologies (Austin, TX, USA). Polyclonal anti-CTSB antibody, polyclonal anti-Beclin-1 antibody, polyclonal anti-ATG3 antibody, polyclonal anti-ATG5 antibody, polyclonal anti-ATG7 antibody, polyclonal anti-ATG12 antibody, polyclonal anti-ATG16 antibody, polyclonal anti-ATG4A antibody, polyclonal anti-ATG4B antibody, polyclonal anti-ATG9B antibody, polyclonal anti-LC3-II antibody were obtained from Abcam Inc. (Cambridge, MA, USA). TRAIL DR5 siRNA, protease inhibitor and other chemicals were purchased from Sigma-Aldrich (St. Louis, MO, USA).

Human breast cancer cell line MDA-MB-231 or MCF-7 (from American Type Culture Collection) was maintained in Leibovitz’s L-15 medium or RPMI-1640 medium, respectively, with 15% fetal bovine serum (FBS), 100 U/ml penicillin and 100 *μ*g/ml streptomycin with 5% CO_2_ at 37 °C. Cells were incubated in 96-wells plate (1.0×10^4^ cells/ml), 6-wells plate (5.0×10^5^ cells/ml), and 100 mm dish (1.5×10^7^ cells/ml). Semi-confluent cells were starved for 24 h in basal medium (with DMSO) without FBS and treated with different compounds.

### SAHA dose-response effects

The dose-response effects of SAHA were assessed using xCELLigence real time cell analyzer (RTCA) SP system according to the manufacturer’s protocol. In brief, 1000 cells were loaded in 16-well plates (E-plate 16 ACEA Biosciences, Inc., San Diego, CA, USA) with 150 *μ*l medium/well. After 24 h incubation, MDA-MB-231 or MCF-7 cells were starved as described above and incubated with various concentrations of SAHA (0, 0.5, 1.0, 2.0, 5.0, 10, 20, and 50 *μ*M) for 24 h. Baseline cell index were calculated for at least two measurements from three replicate experiments. Cell proliferation was monitored for another 48 h.

### TRAIL DR5 siRNA transfection

Just before transfection, MDA-MB-231 or MCF-7 breast cancer cells were grown with Leibovitz’s L-15 medium or RPMI-1640 medium, respectively, without FBS. TRAIL DR5 siRNA transfection was performed using Lipofectamine 3000 reagent following the manufacturer’s recommendations. For each 6-well 6.6 *μ*l siRNA were mixed with 125 *μ*l incomplete culture medium. In another tube 125 *μ*l of incomplete culture medium were mixed with 3.75 *μ*l lipofectamine 3000 by pipetting, kept at RT for 5 min and then added to the diluted siRNA, again mixed by pipetting and kept at RT for another 5 min. Then 1.75 ml of complete culture medium was added to the cells and 250 *μ*l of the prepared siRNA/lipofectamine 3000 solution was added drop-wise followed by gentle shaking of the plate. After 4 h incubation of the cells at 37 °C in a humidified CO_2_ incubator, SAHA or complete culture medium was added, with cells being cultured under the same conditions before RNA and protein were extracted for Real-Time PCR and western blotting.

### Cell viability and apoptosis assay

MDA-MB-231 or MCF-7 cells were plated in 6-well plate. After synchronization with DMSO (basal medium) without FBS for 24 h. The cells were transfected with TRAIL DR5 siRNA as described as above. Then MDA-MB-231 or MCF-7 cells were incubated in complete culture medium containing 5 *μ*M or 10 *μ*M SAHA, respectively, for 24 h.

For cell viability assay, 2×10^5^ of collected cells (50 *μ*l cell suspension) was treated with 450 *μ*l Count and Viability reagent. Muse Count and Viability software module was used to analyze the concentration and percentage of viable cells.

For the apoptotic assay, 1×10^6^ of cells were added with 100 *μ*l of Muse Annexin V and Dead Cell reagent for 20 min at room temperature. Muse Cell Analyzer was determined the percentages of the cells represented by alive, apoptosis and dead population.

### RNA extraction, real-time PCR and real-time PCR array

High Pure RNA Isolation kit was used to extract the total RNA from MDA-MB-231 or MCF-7 cells treated with SAHA and TRAIL DR5 siRNA.

To obtain the first-strand cDNA, transcriptor first strand cDNA synthesis kit was used and cDNA was as a template in real-time PCR reactions with power SYBR green PCR master mix. Real-time PCR analysis was performed using ABI 7500 system and GAPDH was applied as an internal control. The TRAIL DR5 primer sequences for PCR amplification were 5′- GGAACTTTCCGGAATGACAA-3′ (sense) and 5′- GTCACTCCAGGGCGTACAAT-3′ (antisense), GAPDH primer sequences were 5′- GAGTCAACGGATTTGGTCGT-3′ (sense) and 5′- GACAAGCTTCCCGTTCTCAG-3′ (antisense).

Exprofile human autophagy Gene qPCR array kit was employed to describe related mRNA expression. Relative changes of gene expression in the array were calculated using the 2^−ΔΔCt^ (threshold cycle) method.

### Western blot analysis

MDA-MB-231 or MCF-7 cells were homogenizated in NP40 cell lysis buffer contained 0.1 mg/ml protease inhibitor, 1 mM PMSF. BCA protein assay kit was used to measure the protein concentrations. Twenty micrograms of proteins was loaded per lane on SDS-polyacrylamide gels and transferred to PVDF membranes. Western blot analyzes were performed to assess the levels for autophagy-related factors and the level of β-actin was used as loading controls. Protein bands were detected using supersignal west pico plus chemiluminescent substrate and exposed on DNR MF-Chemi Bio-imaging systems.

### LC3-II fluorescence microscopy

TRAIL DR5 transfected cells of MDA-MB-231 or MCF-7 were incubated with SAHA in 6-well plate as indicated above. The cells were fixed with 4% formaldehyde for 10 min at room temperature and blocked with buffer solution containing 10% goat serum, 0.3 M glycine, 1% BSA and 0.1% tween for 2 h at room temperature. LC3-II antibody solution was incubate with cells overnight at 4 °C. A DyLight 488 fluoresence antibody (1 : 200 dilution) was used for 1 h incubation and nuclei were counterstained with DAPI dye for another 10 min. The cells were imaged and autophagy signals were visualized by Leica DMI6000 B microscope with ×10 and ×63 magnification.

### CTSB activity assay

MDA-MB-231 or MCF-7 cells were collected at the indicated above. Cellular lysate was gathered and the activity of CTSB was performed using the colorimetric ELISA assay according to the manufacturer’s instruction. The enzymatic activity of pro-CTSB was detected by a microplate reader at 450 nm.

### Human MAPK antibody array

First, ~2×10^7^ cells with SAHA and TRAIL DR5 siRNA treatment were solubilized in lysis buffer. The lysates were resuspended gently at 4 °C for 30 min and centrifuged at 14 000 g for 5 min. Protein concentrations of the resulting lysates were measured using a BCA protein assay kit. MAPK antibody array was used to screen the activity changes involved in autophagy-related signaling pathways. A amount of 400 *μ*g of prepared cell lysates were incubated with reconstituted detection antibody cocktail at room temperature for 1 h. The sample/antibody mixtures were added to each well of the prepared dish for incubation overnight at 4 °C with gentle shaking. After washing, the membranes were incubated with 2 ml streptavidin-HRP for 1 h with gently shaking. Next, membrane intensity was obtained using chemiluminescence and pixel densities were analyzed using Gelpro Analyzer software (Media Cybernetics, Rockville, MD, USA). Densities were measured as a percentage of the positive controls included on each membrane. After subtracting background signals and normalization to positive controls, comparison of signal intensities among array images can be used to determine relative differences in expression levels of each protein between groups.

### Data analysis

Student’s *t*-test was used for data analysis. Data are presented as mean±S.E.M. Values for *P*<0.05 were considered statistically significant. The model included the main effects of treatments and replicates.

## Conclusion

In summary, the anti-tumor effect of SAHA has been confirmed, but its full target has not been fully elucidated. This study is the first study to clarify the SAHA functions in autophagy through effective regulations of TRAIL DR5 and CTSB and thereby inhibit the proliferation of breast cancer cells and hinder the occurrence of breast cancer.

## Publisher’s note

Springer Nature remains neutral with regard to jurisdictional claims in published maps and institutional affiliations.

## References

[bib1] Siegel RL, Miller KD, Jemal A. Cancer statistics. CA Cancer J Clin 2016; 67: 7–30. 10.3322/caac.21332 26742998

[bib2] Lim E, Metzger-filho O, Winer EP. The natural history of hormone receptor-positive breast cancer. Oncology 2012; 26: 688–694. 22957400

[bib3] Marks PA. Discovery and development of SAHA as an anticancer agent. Oncogene 2007; 26: 1351–1356. 17322921 10.1038/sj.onc.1210204

[bib4] Feng X, Han H, Zou D, Zhou J, Zhou W. Suberoylanilide hydroxamic acid-induced specific epigenetic regulation controls Leptin-induced proliferation of breast cancer cell lines. Oncotarget 2017; 8: 3364–3379. 27926517 10.18632/oncotarget.13764 PMC5356888

[bib5] Brunetti G, Oranger A, Carbone C, Mori G, Sardone FR, Mori C et al. Osteoblasts display different responsiveness to TRAIL-induced apoptosis during their differentiation process. Cell Biochem Biophys 2013; 67: 1127–1136. 23677859 10.1007/s12013-013-9616-6

[bib6] Portanova P, Notaro A, Pellerito O, Sabella S, Giuliano M, Calvaruso G. Notch inhibition restores TRAIL-mediated apoptosis via AP1-dependent upregulation of DR4 and DR5 TRAIL receptors in MDA-MB-231 breast cancer cells. Int J Oncol 2013; 43: 121–130. 23686163 10.3892/ijo.2013.1945

[bib7] Amm HM, Oliver PG, Lee CH, Li Y, Buchsbaum DJ. Combined modality therapy with TRAIL or agonistic death receptor antibodies. Cancer Biol Ther 2011; 11: 431–449. 21263219 10.4161/cbt.11.5.14671 PMC3087899

[bib8] Kischkel FC, Lawrence DA, Chuntharapai A, Schow P, Kim KJ, Ashkenazi A. Apo2L/TRAIL-dependent recruitment of endogenous FADD and caspase-8 to death receptors 4 and 5. Immunity 2000; 12: 611–620. 10894161 10.1016/s1074-7613(00)80212-5

[bib9] Shin GC, Kang HS, Lee AR, Kim KH. Hepatitis B Virus-triggered Autophagy Targets TNFRSF10B/Death Receptor 5 for Degradation to Limit TNFSF10/TRAIL Response. Autophagy 2016; 12: 2451–2466. 27740879 10.1080/15548627.2016.1239002 PMC5173271

[bib10] Kelley RF, Totpal K, Lindstrom SH, Mathieu M, Billeci K, Deforge L et al. Receptor-selective mutants of apoptosis-inducing ligand 2/tumor necrosis factor-related apoptosis-inducing ligand reveal a greater contribution of death receptor (DR) 5 than DR4 to apoptosis signaling. J Biol Chem 2005; 280: 2205–2212. 15520016 10.1074/jbc.M410660200

[bib11] Akpinar B, Safarikova B, Laukova J, Debnath S, Vaculova AH, Zhivotovsky B et al. Aberrant DR5 transport through disruption of lysosomal function suggests a novel mechanism for receptor activation. Oncotarget 2016; 7: 58286–58301. 27506940 10.18632/oncotarget.11073 PMC5295431

[bib12] Zhu J, Zhou Q, Tan S. Targeting miRNAs associated with surface expression of death receptors to modulate TRAIL resistance in breast cancer. Cancer Lett 2016; 383: 154–160. 27693456 10.1016/j.canlet.2016.09.021

[bib13] Park SH, Chi GY, Eom HS, Kim GY, Hyun JW, Kim WJ et al. Role of autophagy in apoptosis induction by methylene chloride extracts, of Mori cortex in NCI-H460 human lung carcinoma cells. Int J Oncol 2012; 40: 1929–1940. 22367066 10.3892/ijo.2012.1386

[bib14] Kondo Y, Kanzawa T, Sawaya R, Kondo S. The role of autophagy in cancer development and response to therapy. Nat Rev Cancer 2005; 5: 726–734. 16148885 10.1038/nrc1692

[bib15] Maiuri MC, Zalckvar E, Kimchi A, Kroemer G. Self-eating and self-killing: crosstalk between autophagy and apoptosis. Nat Rev Mol Cell Biol 2007; 8: 741–752. 17717517 10.1038/nrm2239

[bib16] Reksodiputro AH. Autophagy. Acta Med Indones 2007; 39: 151–152. 18376517

[bib17] Bröker LE, Kruyt FA, Giaccone G. Cell death independent of caspases: a review. Clin Cancer Res 2005; 11: 3155–3162. 15867207 10.1158/1078-0432.CCR-04-2223

[bib18] Lamore SD, Wondrak GT. Autophagic-lysosomal dysregulation downstream of cathepsin B inactivation in human skin fibroblasts exposed to UVA. Photochem Photobiol Sci 2012; 11: 163–172. 21773629 10.1039/c1pp05131h PMC4089038

[bib19] Gondi CS, Rao JS. Cathepsin B as a cancer target. Expert Opin Ther Targets 2013; 17: 281–291. 23293836 10.1517/14728222.2013.740461 PMC3587140

[bib20] Bhoopathi P, Chetty C, Gujrati M, Dinh DH, Rao JS, Lakka S. Cathepsin B facilitates Autophagy mediated apoptosis in SPARC overexpressed primitive neuroectodermal tumor cells. Cell Death Differ 2010; 17: 1529–1539. 20339379 10.1038/cdd.2010.28 PMC3025815

[bib21] Man SM, Kanneganti TD. Regulation of lysosomal dynamics and autophagy by CTSB/cathepsin B. Autophagy 2016; 12: 2504–2505. 27786577 10.1080/15548627.2016.1239679 PMC5173259

[bib22] Aggarwal N, Sloane BF. Cathepsin B: multiple roles in cancer. Proteomics Clin Appl 2014; 8: 427–437. 24677670 10.1002/prca.201300105 PMC4205946

[bib23] Konduri SD, Yanamandra N, Siddique K, Joseph A, Dinh DH, Olivero WC et al. Down-regulation of cathepsin B expression impairs the invasive and tumorigenic potential of human glioblastoma cells. Oncogene 2002; 21: 8705–8712. 12483523 10.1038/sj.onc.1205949

[bib24] Lakka SS, Gondi CS, Yanamandra N, Olivero WC, Dinh DH, Gujrati M et al. Inhibition of cathepsin B and MMP-9 gene expression in glioblastoma cell line via RNA interference reduces tumor cell invasion, tumor growth and angiogenesis. Oncogene 2004; 23: 4681–4689. 15122332 10.1038/sj.onc.1207616

[bib25] Lu NN, Liu J, Tian Y, Liao MH, Wang H, Lu YM et al. Atg5 deficit exaggerates the lysosome formation and cathepsin B activation in mice brain after lipid nanoparticles injection. Nanomedicine 2014; 10: 1843–1852. 24768629 10.1016/j.nano.2014.03.019

[bib26] Han F, Chen YX, Lu YM, Huang JY, Zhang GS, Tao RR et al. Regulation of the ischemia-induced autophagy-lysosome processes by nitrosative stress in endothelial cells. J Pineal Res 2011; 51: 124–135. 21392095 10.1111/j.1600-079X.2011.00869.x

[bib27] Klionsky DJ, Eskelinen EL, Deretic V. Autophagosomes, phagosomes, autolysosomes, phagolysosomes, autophagolysosomes… wait, I'm confused. Autophagy 2014; 10: 549–551. 24657946 10.4161/auto.28448 PMC4091142

[bib28] Padman BS, Nguyen TN, Lazarou M. Autophagosome formation and cargo sequestration in the absence of LC3/GABARAPs. Autophagy 2017; 13: 772–774. 28165849 10.1080/15548627.2017.1281492 PMC5388231

[bib29] Wang SH, Shih YL, Ko WC, Wei YH, Shih CM. Cadmium-induced autophagy and apoptosis are mediated by a calcium signaling pathway. Cell Mol Life Sci 2008; 65: 3640–3652. 18850067 10.1007/s00018-008-8383-9 PMC11131605

[bib30] Xie SQ, Li Q, Zhang YH, Wang JH, Mei ZH, Zhao J et al. NPC-16, a novel naphthalimide-polyamine conjugate, induced apoptosis and autophagy in human hepatoma HepG2 cells and Bel-7402 cells. Apoptosis 2011; 16: 27–34. 20809291 10.1007/s10495-010-0537-1

[bib31] Zhou W, Feng X, Han Han, Guo S, Wang G. Synergistic effects of combined treatment with histone deacetylase inhibitor suberoylanilide hydroxamic acid and TRAIL on human breast cancer cells. Sci Rep 2016; 6: 28004. 27292433 10.1038/srep28004 PMC4904277

[bib32] Lauricella M, Ciraolo A, Carlisi D, Vento R, Tesoriere G. SAHA/TRAIL combination induces detachment and anoikis of MDA-MB231 and MCF-7 breast cancer cells. Biochimie 2012; 94: 287–299. 21835222 10.1016/j.biochi.2011.06.031

[bib33] Ni Z, Gong Y, Dai X, Ding W, Wang B, Gong H et al. AU4S: a novel synthetic peptide to measure the activity of ATG4 in living cells. Autophagy 2015; 11: 403–415. 25831015 10.1080/15548627.2015.1009773 PMC4502827

[bib34] Yu ZQ, Ni T, Hong B, Wang HY, Jiang FJ, Zou S et al. Dual roles of Atg8-PE deconjugation by Atg4 in autophagy. Autophagy 2012; 8: 883–892. 22652539 10.4161/auto.19652 PMC3427254

[bib35] Xie Z, Klionsky DJ. Autophagosome formation: core machinery and adaptations. Nat Cell Biol 2007; 9: 1102–1109. 17909521 10.1038/ncb1007-1102

[bib36] Satoo K, Noda NN, Kumeta H, Fujioka Y, Mizushima N, Ohsumi Y et al. The structure of Atg4B-LC3 complex reveals the mechanism of LC3 processing and delipidation during autophagy. EMBO J 2009; 28: 1341–1350. 19322194 10.1038/emboj.2009.80 PMC2683054

[bib37] Kirisako T, Ichimura Y, Okada H, Kabeya Y, Mizushima N, Yoshimori T et al. The Reversible Modification Regulates the Membrane-Binding State of Apg8/Aut7 Essential for Autophagy and the Cytoplasm to Vacuole Targeting Pathway. J Cell Biol 2000; 151: 263–276. 11038174 10.1083/jcb.151.2.263 PMC2192639

[bib38] Xie Z, Nair U, Klionsky DJ. Atg8 controls phagophore expansion during autophagosome formation. Mol Biol Cell 2008; 19: 3290–3298. 18508918 10.1091/mbc.E07-12-1292 PMC2488302

[bib39] Pei D, Zhang W, Sun H, Wei X, Yue J, Wang H. Identification of autophagy-related genes ATG4 and ATG8 from wheat (Triticum aestivum L.) and profiling of their expression patterns responding to biotic and abiotic stresses. Plant Cell Rep 2014; 33: 1697–1710. 24996626 10.1007/s00299-014-1648-x

[bib40] Seo E, Woo J, Park E, Bertolani SJ, Siegel JB, Choi D et al. Comparative analyses of ubiquitin-like ATG8 and cysteine protease ATG4 autophagy genes in the plant lineage and cross-kingdom processing of ATG8 by ATG4. Autophagy 2016; 12: 2054–2068. 27540766 10.1080/15548627.2016.1217373 PMC5103345

[bib41] Sarbassov DD, Ali SM, Sabatini DM. Growing roles for the mTOR pathway. Curr Opin Cell Biol 2005; 17: 596–603. 16226444 10.1016/j.ceb.2005.09.009

[bib42] Yu Y, Hou L, Song H, Xu P, Sun Y, Wu K. Akt/AMPK/mTOR pathway was involved in the autophagy induced by vitamin E succinate in human gastric cancer SGC-7901 cells. Mol Cell Biochem 2017; 424: 173–183. 27796683 10.1007/s11010-016-2853-4

